# MRSA Infections in HIV-Infected People Are Associated with Decreased MRSA-Specific Th1 Immunity

**DOI:** 10.1371/journal.ppat.1005580

**Published:** 2016-04-19

**Authors:** Netanya S. Utay, Annelys Roque, J. Katherina Timmer, David R. Morcock, Claire DeLeage, Anoma Somasunderam, Amy C. Weintrob, Brian K. Agan, Jacob D. Estes, Nancy F. Crum-Cianflone, Daniel C. Douek

**Affiliations:** 1 Division of Infectious Diseases, Department of Internal Medicine, University of Texas Medical Branch, Galveston, Texas, United States of America; 2 Human Immunology Section, Vaccine Research Center, National Institutes of Allergy and Infectious Diseases, National Institutes of Health, Bethesda, Maryland, United States of America; 3 AIDS and Cancer Virus Program, Leidos Biomedical Research, Inc., Frederick National Laboratory for Cancer Research, Frederick, Maryland, United States of America; 4 Infectious Disease Clinical Research Program (IDCRP), Department of Preventive Medicine and Biostatistics, Uniformed Services University of the Health Sciences (USUHS), Bethesda, Maryland, United States of America; 5 Walter Reed National Military Medical Center, Bethesda, Maryland, United States of America; 6 Henry M. Jackson Foundation for the Advancement of Military Medicine, Bethesda, Maryland, United States of America; 7 Infectious Disease Clinic, Naval Medical Center San Diego, San Diego, California, United States of America; Emory University, UNITED STATES

## Abstract

People with HIV infection are at increased risk for community-acquired methicillin-resistant *Staphylococcus aureus* (CA-MRSA) skin and soft tissue infections (SSTIs). Lower CD4 T-cell counts, higher peak HIV RNA levels and epidemiological factors may be associated with increased risk but no specific immune defect has been identified. We aimed to determine the immunologic perturbations that predispose HIV-infected people to MRSA SSTIs. Participants with or without HIV infection and with MRSA SSTI, MRSA colonization or negative for MRSA were enrolled. Peripheral blood and skin biopsies from study participants were collected. Flow cytometry, flow cytometry with microscopy, multiplex assays of cell culture supernatants and immunohistochemistry were used to evaluate the nature of the immune defect predisposing HIV-infected people to MRSA infections. We found deficient MRSA-specific IFNγ^+^ CD4 T-cell responses in HIV-infected people with MRSA SSTIs compared to MRSA-colonized participants and HIV-uninfected participants with MRSA SSTIs. These IFNγ^+^ CD4 T cells were less polyfunctional in HIV-infected participants with SSTIs compared to those without SSTIs. However, IFNγ responses to cytomegalovirus and *Mycobacterium avium* antigens and MRSA-specific IL-17 responses by CD4 T cells were intact. Upon stimulation with MRSA, peripheral blood mononuclear cells from HIV-infected participants produced less IL-12 and IL-15, key drivers of IFNγ production. There were no defects in CD8 T-cell responses, monocyte responses, opsonization, or phagocytosis of *Staphylococcus aureus*. Accumulation of CD3 T cells, CD4 T cells, IL-17^+^ cells, myeloperoxidase^+^ neutrophils and macrophage/myeloid cells to the skin lesions were similar between HIV-infected and HIV-uninfected participants based on immunohistochemistry. Together, these results indicate that MRSA-specific IFNγ^+^ CD4 T-cell responses are essential for the control of initial and recurrent MRSA infections in HIV-infected people.

## Author Summary

Methicillin-resistant *Staphylococcus aureus* (MRSA) causes infections of the skin and associated tissue. HIV-infected people are at increased risks of these infections for unclear reasons. We recruited participants with or without HIV infection who had a MRSA skin or soft tissue infection, MRSA colonization, or neither, and obtained blood and skin biopsy samples. We found that HIV-infected people with MRSA infections mounted insufficient responses upon stimulation with MRSA. These participants had decreased proportions of cells producing the cytokine IFNγ and decreased function of IFNγ-producing cells in response to in vitro stimulation with MRSA, but their responses to other agents such as cytomegalovirus were intact. The decrease in IFNγ-producing cells may be due to decreased production of upstream drivers of IFNγ production, such as IL-12 and IL-15. Nonetheless, responses in the skin were not affected. As IFNγ facilitates T-cell proliferation and response to antigens, and macrophage and neutrophil responses, this antigen-specific defect may have widespread effects. Together, our data suggest that MRSA-specific IFNγ responses may be essential for effective prevention against future infections, including vaccine development.

## Introduction

Community-acquired methicillin-resistant *Staphylococcus aureus* (CA-MRSA) emerged as a major cause of skin and soft tissue infections (SSTIs) in the 1990s. SSTIs manifest as cellulitis, abscesses, folliculitis, furuncles and carbuncles [1]. MRSA colonizes 8.8% of HIV-infected persons in North America [2]. HIV-infected people are 18-fold more likely to have CA-MRSA infections than HIV-uninfected people and twice as likely to have recurrences [3]. Risk factors for CA-MRSA infection include higher peak HIV RNA levels, lower nadir and current CD4 T-cell counts, no antiretroviral therapy, same-sex intercourse among men, multiple sexual partners, recent sexually transmitted infections, close contact with MRSA-infected persons and injection drug use [4]. However, CA-MRSA SSTIs still affect HIV-infected participants with relatively high CD4 T-cell counts (mean 430 cells/mm^3^) [4], suggesting an antigen-specific rather than global immune defect.

The reason that lower CD4 T-cell counts may predispose HIV-infected people to MRSA SSTIs remains unclear. Mouse studies suggest that interferon γ (IFNγ) contributes to protection against *S*. *aureus* infection [5, 6]. IFNγ is used for refractory *S*. *aureus* infections in chronic granulomatous disease (CGD) [7]. However, mice deficient in IL-17 have more severe *S*. *aureus* SSTIs [8]. People with hyperimmunoglobulin E syndrome, who lack Th17 cells, are predisposed to *S*. *aureus* abscesses [9]. IL-17 stimulates antimicrobial peptide production and neutrophil recruitment. Importantly, Th17 cells are rapidly depleted from the gastrointestinal tract upon HIV infection [10]. We hypothesized that MRSA SSTIs are increased in HIV-infected persons due to defects in MRSA-specific CD4 T-cell responses.

## Results

### Baseline characteristics

Fifty-two participants were recruited (Table 1). HIV-infected participants were significantly older than HIV-uninfected participants (median age 46.0 versus 25.5 years, *P*<0.0001) with lower median CD4:CD8 ratios (1.1 versus 2.2, *P*<0.0001). Gender distribution, history of MRSA infection, recent hospitalization or ER visit, and for HIV-infected participants, years of HIV seropositivity, CD4 T-cell counts, CD4 T-cell nadir, plasma HIV RNA levels and ART use did not differ among groups.

**Table 1:** Baseline characteristics.

Characteristic	Total	HIV+	HIV+	HIV+	HIV-	HIV-	HIV-
	Cohort	MRSA Infected	MRSA Colonized	MRSA-	MRSA Infected	MRSA Colonized	MRSA-
**N**	52	8	7	11	10	5	11
**Age, years**	36.0^A^	44	40	46	25	24	30
** **	(25.0–48.0)	(34–53)	(24–75)	(28–57)	(19–52)	(22–36)	(21–76)
**Gender: Male**	42 (81%)	8 (100%)	6 (86%)	10 (91%)	8 (80%)	3 (60%)	7 (64%)
**Female**	10 (19%)		1 (14%)	1 (9%)	2 (20%)	2 (40%)	4 (36%)
**History of MRSA infection**	21 (40%)	7 (88%)	3 (43%)	0 (0%)	9 (90%)	0 (0%)	0 (0%)
**Hospitalization or ER visit (last 12 months)**	17	8 (100%)	7 (100%)	2 (18%)	10 (100%)	2 (40%)	4 (36%)
**CD4 count, cells/μl**	521	342	600	525			
** **	(9–1432)	(9–810)	(215–1432)	(239–913)			
**CD4 nadir, cells/μl**	195	80	264	176			
** **	(0–568)	(0–358)	(107–453)	(15–568)			
**CD4/CD8**	1.7	1.0	1.2	1.3	1.9	2.2	2.6
	(0.01–7.9)	(0.01–1.8)	(0.3–2.2)	(0.5–1.8)	(0.7–5.2)	(1.0–2.6)	(1.5–7.9)
**HIV RNA (log 10 copies/ml)**	1.7	1.7	1.7	1.7			
	(1.7–1.9)	(1.7–5.6)	(1.7–2.3)	(1.7–4.8)			
**% with HIV RNA <50 copies/ml**	20 (77%)	5 (63%)	6 (86%)	9 (82%)			
**Taking ART**	25	8 (100%)	7 (100%)	10 (91%)			

^A^ All values are N (%) or median (range)

### Antigen-specific T-cell responses

First, we evaluated whether circulating CD4 T-cell subset differences contributed to increased susceptibility to MRSA SSTIs. HIV-infected participants with MRSA SSTIs had higher frequencies of central memory-like CD27^+^CD45RO^+^ CD4 T cells than HIV-infected MRSA-negative participants (*P* = 0.02, S1A Fig) and effector memory CD27^-^ (*P* = 0.009, S1B Fig) and terminally differentiated CD57^+^ CD4 T cells (*P* = 0.03, S1C Fig) than HIV-uninfected participants with MRSA SSTIs. Thus, HIV-infected participants with MRSA SSTIs did not have fewer memory CD4 T cells but did have more terminally differentiated CD4 T cells.

**Fig 1:**
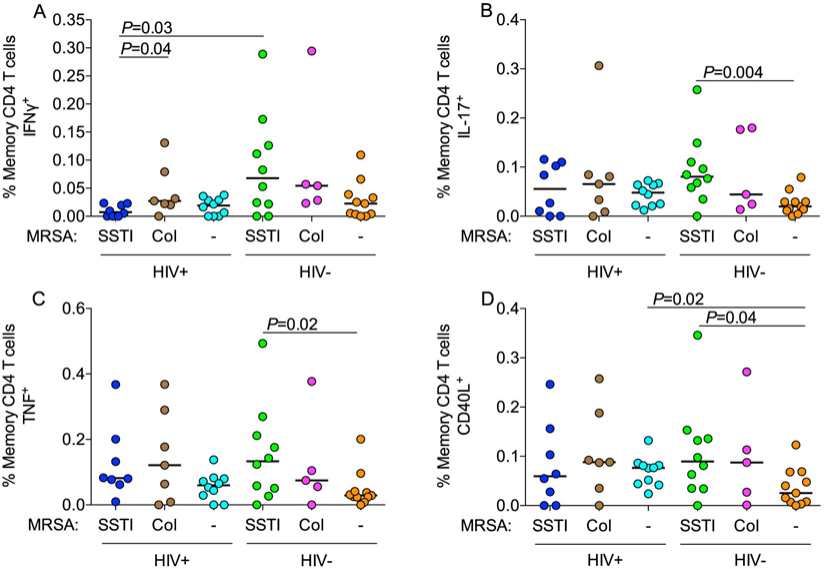
MRSA-specific memory CD4 T cells. Flow cytometry analysis of MRSA-specific memory (CD27^+^CD45RO^+^ or CD27^-^) CD4 T-cell responses in HIV-infected or HIV-uninfected participants with MRSA SSTI, colonization or neither. Frequency of memory CD4 T cells producing (A) IFNγ, (B) IL-17, (C) TNF and (D) CD40L. For all figures, horizontal lines indicate medians. *P*-values were calculated using the Mann-Whitney *U* test.

Next, we measured MRSA-specific CD4 T-cell responses. Among HIV-infected participants, the frequencies of MRSA-specific IFNγ^+^, IL-17^+^, CD40L^+^, TNF^+^, IL-2^+^ or IL-22^+^ memory CD4 T cells did not differ significantly between MRSA SSTI and MRSA-negative groups (Fig 1). HIV-infected participants with MRSA SSTIs had lower MRSA-specific IFNγ^+^ memory CD4 T-cell frequencies compared to MRSA-colonized participants (0.007% vs 0.03%, *P* = 0.04; Fig 1A). Among HIV-uninfected participants, the MRSA SSTI group had higher MRSA-specific IL-17^+^ (0.08% vs. 0.02%, *P* = 0.004, Fig 1B), TNF^+^ (0.13% vs 0.03%, *P* = 0.02, Fig 1C) and CD40L^+^ (0.09% vs. 0.03%, *P* = 0.04, Fig 1D) memory CD4 T-cell frequencies than MRSA-negative participants. The MRSA-specific IFNγ^+^ (0.07% vs. 0.02%, *P* = 0.14; Fig 1A), IL-2^+^ (0.08% vs. 0.01%, *P* = 0.05) and IL-22^+^ (0.04% vs. 0.001%, *P* = 0.12) CD4 T-cell frequencies were not significantly higher in HIV-uninfected participants with MRSA SSTIs than MRSA-negative participants. However, HIV-infected participants with MRSA SSTIs had significantly lower MRSA-specific IFNγ^+^ memory CD4 T-cell frequencies compared to HIV-uninfected participants with MRSA SSTIs (0.007% vs 0.07%, *P* = 0.03, Fig 1A). The frequencies of other cytokine-producing MRSA-specific memory CD4 T cells or memory CD8 T cells did not differ significantly. Thus, HIV-infected participants with MRSA SSTIs had deficient MRSA-specific IFNγ^+^ memory CD4 T-cell responses.

To further characterize MRSA-specific responses, we analyzed the polyfunctionality of memory CD4 T cells. HIV-infected participants with MRSA SSTIs had fewer MRSA-specific polyfunctional cells (CD40L, IL-2, TNF, IL-17 and/or IFNγ) than HIV-infected MRSA-negative participants (*P* = 0.047, S2A Fig). HIV-infected participants with MRSA SSTIs had fewer polyfunctional MRSA-specific IFNγ^+^ cells than HIV-uninfected participants with MRSA SSTIs (*P* = 0.02 for IFNγ^+^TNF^+^IL-2^+^, *P* = 0.03 for IFNγ^+^TNF^+^IL-2^-^ and *P* = 0.01 for IFNγ^+^TNF^-^IL-2^+^ memory CD4 T cells; S2B Fig). MRSA-specific polyfunctionality did not differ significantly among other groups. In sum, MRSA-specific polyfunctional CD4 IFNγ^+^ T-cell responses were deficient in HIV-infected participants with MRSA SSTIs.

To determine the extent of this defect, we measured responses to other stimuli. No significant differences in the frequencies of IFNγ^+^ or IL-17^+^ memory CD4 T cells were detected upon stimulation with cytomegalovirus (CMV), *Candida albicans* or *Mycobacterium avium* antigens (S3A–S3C Fig). However, HIV-infected participants with MRSA SSTIs had decreased IFNγ^+^ but not IL-17^+^ memory CD4 T-cells frequencies upon *Staphylcoccus* enterotoxin B stimulation compared to HIV-uninfected participants with MRSA SSTIs (SEB; 6.9% vs. 11.5%, *P* = 0.02, S3D Fig). Thus, the decreased IFNγ^+^ responses were MRSA-specific.

Next, we evaluated whether loss of MRSA-specific responses correlated with MRSA SSTI development. Flow cytometry analysis showed no significant differences in frequencies of MRSA-specific IFNγ^+^ or IL-17^+^ CD4 T cells among PBMCs collected prior to enrollment (T1, median 244 days, range 133–391 days), at enrollment (T2) and after enrollment (T3, median 237 days, range 140–404 days) (S4A–S4C Fig). However, the circulating CCR4^+^ CD4 T-cell frequencies decreased between pre-SSTI and SSTI time points in HIV-infected participants with MRSA SSTI (23.1% to 16.2%, *P* = 0.06; S4D–S4F Fig) but not in HIV-infected MRSA-colonized or MRSA-negative groups. In sum, HIV-infected participants with MRSA SSTIs had decreased MRSA-specific IFNγ^+^ and CCR4^+^ CD4 T-cell frequencies.

### Multiplex cytokine analysis of cell culture supernatants

**Fig 2:**
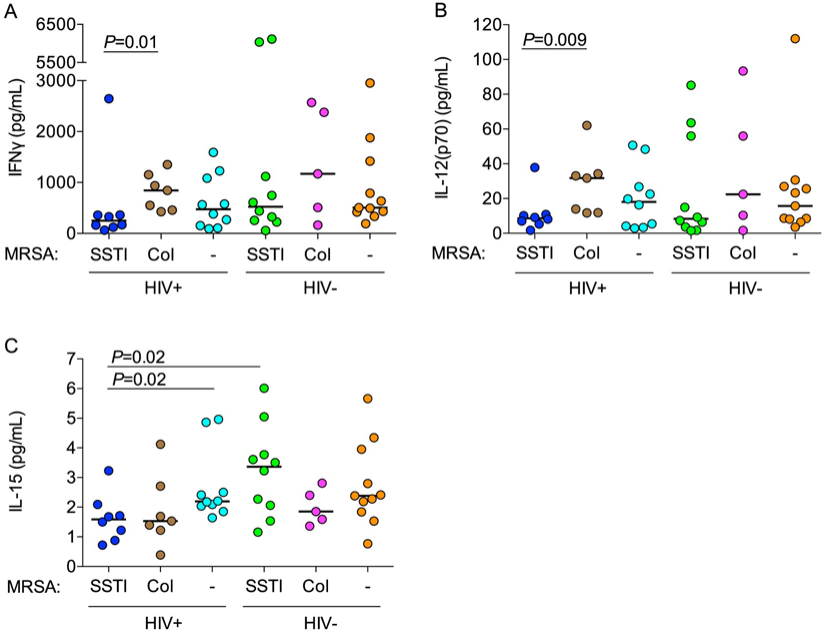
Cytokine concentrations released into supernatant. Supernatant concentrations of IFNγ (A) and drivers of IFNγ production, IL-12 (B) and IL-15 (C), after MRSA stimulation of PBMCs were assayed using the Luminex platform. *P*-values were calculated using the Mann-Whitney *U* test.

To evaluate comprehensively the responsiveness of MRSA-specific PBMCs, we measured cytokines released into the supernatant after stimulation with heat-killed MRSA. Indeed, PBMCs from HIV-infected participants with MRSA SSTIs produced less IFNγ and IL-12p70 than PBMCs from HIV-infected, MRSA-colonized participants (250 vs. 844 pg/ml, *P* = 0.01, Fig 2A; 8.7 vs. 31.8 pg/ml, *P* = 0.009, Fig 2B). IL-15 production was also significantly lower in HIV-infected MRSA SSTI participants compared to HIV-infected MRSA-negative (1.59 vs. 2.20 pg/ml, *P* = 0.02) and HIV-uninfected MRSA SSTI groups (1.59 vs. 3.37 pg/ml, *P* = 0.02; Fig 2C). Production of IL-17 and IL-10 did not differ significantly among groups, nor did IFNγ or IL-12p70 production in response to stimulation with CMV or *C*. *albicans* antigens. In sum, HIV-infected participants with MRSA SSTIs had decreased MRSA-specific IFNγ, IL-12p70 and IL-15 production.

### Phagocytosis

To assess phagocytosis, first we assessed whether decreased monocyte responses contributed to the increased susceptibility to MRSA SSTIs in HIV infection by flow cytometry analysis. The HIV-infected MRSA SSTI group had significantly higher frequencies of circulating CD14^+^ monocytes (2.39% vs. 0.62%, *P* = 0.009; S5A Fig) and CD14^+^ monocytes producing IL-1β (46.75% vs. 13.00%, *P* = 0.02; S5B Fig) or TNF (45.70% vs. 13.90%, *P* = 0.02; S5C Fig) after incubation with MRSA compared to HIV-infected MRSA-negative participants. The frequencies of CD14^+^ monocytes producing IL-1β or TNF after incubation with MRSA were similar between HIV-uninfected participants with MRSA SSTIs and MRSA-negative participants. The HIV-infected MRSA SSTI group also had higher, although not statistically significant, frequencies of circulating CD14^+^ monocytes (2.39% vs. 0.69%, *P* = 0.08; S5A Fig) and CD14^+^ monocytes producing IL-1β (46.75% vs. 21.90%, *P* = 0.12; S5B Fig) or TNF (45.70% vs. 24.70%, *P* = 0.20; S5C Fig) compared to HIV-uninfected MRSA SSTI participants, but these differences were not statistically significant. Thus, monocyte frequency and cytokine production increased with MRSA SSTI only in HIV-infected participants.

Next, we evaluated whether defective phagocytosis by monocytes might contribute to increased susceptibility to MRSA SSTI using Amnis Imagestream. The frequencies of CD14^+^ cells with phagocytosed fluorescently-labeled *S*. *aureus* were not significantly higher in HIV-infected participants with MRSA SSTI compared to HIV-uninfected participants with MRSA SSTI (41.7% vs. 16.7%, *P* = 0.79; S6A and S6B Fig). HIV-uninfected participants with SSTI had significantly lower CD14^+^-cell frequencies that phagocytosed >1 bacterium compared to HIV-uninfected MRSA-negative participants (9.5% vs. 23.6%, *P* = 0.04; S6C Fig). In sum, compromised monocyte phagocytosis did not increase susceptibility to MRSA SSTIs.

To test whether defective opsonization of neutrophils predisposed HIV-infected participants to MRSA SSTIs, healthy donor neutrophils were incubated with fluorescently labeled *S*. *aureus* after opsonization with the participants' plasma. The mean fluorescent intensity (MFI) of *S*. *aureus* was significantly higher in HIV-infected participants with MRSA SSTIs (32,218) or MRSA colonization (33,678) compared to HIV-infected MRSA-negative participants (25,038, *P* = 0.03 or 0.02, respectively; S6D Fig). HIV-uninfected participants with MRSA SSTIs had higher *S*. *aureus* MFIs compared to HIV-uninfected MRSA-negative participants (34,341 vs. 29,447), but this did not reach statistical significance. There were no significant differences between HIV-infected and HIV-uninfected participants with MRSA SSTIs or colonization. Thus, defective opsonization does not account for the increased susceptibility of HIV-infected participants to MRSA SSTIs.

### Histological evaluation of skin biopsies

**Fig 3:**
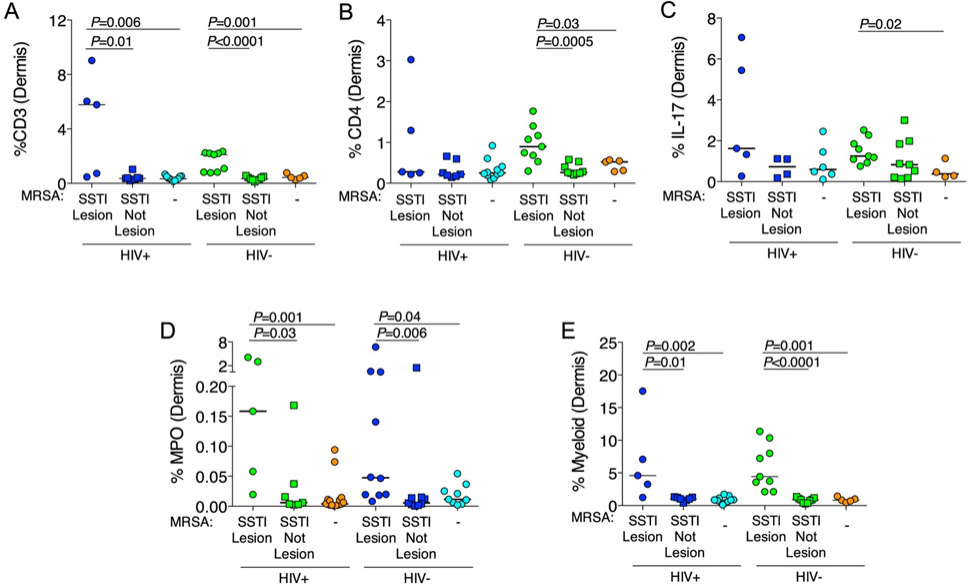
Histological evaluation of skin biopsies. Skin biopsies were obtained from MRSA SSTI participants at the infection site (SSTI lesion) or a distant site (SSTI Not Lesion), or MRSA-negative participants (-). (A) Abundance of CD3. (B) Abundance of CD4. (C) Abundance of IL-17. (D) Abundance of neutrophils (myeloperoxidase^+^ [MPO]). (E) Abundance of macrophage/myeloid cells (CD68^+^ and/or CD163^+^). *P*-values were calculated using the Mann-Whitney *U* test.

Because peripheral blood may not reflect the immunological perturbations in tissue, we evaluated T-cell abundance in skin biopsies. CD3^+^ T-cell abundance was significantly higher in lesional sites from HIV-infected participants compared to non-lesional sites (5.78% vs. 0.36%, *P* = 0.01) and to biopsies from HIV-infected MRSA-negative participants (5.78% vs. 0.33%, *P* = 0.006). CD3^+^ T-cell abundance did not differ between the non-lesional sites and biopsies from HIV-infected MRSA-negative participants (Fig 3A). Similarly, among HIV-uninfected participants with MRSA SSTI, CD3^+^ T-cell abundance was significantly higher in lesional compared to non-lesional sites (2.11% vs. 0.34%, *P<*0.0001) and to biopsies from MRSA-negative participants (2.11% vs. 0.43%, *P* = 0.001), but CD3^+^ T-cell abundance did not differ between the non-lesional sites and biopsies from HIV-uninfected MRSA-negative participants. Among HIV-infected participants, CD4^+^ T-cell abundance did not differ significantly among lesional and non-lesional biopsies and MRSA-negative participants (Fig 3B). In contrast, in HIV-uninfected participants, CD4^+^ T-cell abundance was significantly higher in lesional sites compared to non-lesional sites (0.90% vs. 0.26%, *P* = 0.0005) and to biopsies from MRSA-negative participants (0.90% vs. 0.52%, *P* = 0.03). IL-17^+^-cell abundance was significantly higher in lesional sites of HIV-uninfected participants with MRSA SSTIs compared to biopsies from MRSA-negative participants (1.25% vs. 0.38%, *P* = 0.02) (Fig 3C). IL-17^+^-cell abundance was also higher in lesional sites of HIV-infected participants with MRSA SSTIs compared to biopsies from MRSA-negative participants, but this finding did not reach statistical significance. No significant differences in CD3, CD4 or IL-17 abundance were observed between HIV-infected and HIV-uninfected participants. In sum, lesional sites had more lymphocyte, CD4 and IL-17 infiltration compared to non-lesional sites and biopsies from MRSA-uninfected participants.

Next, we evaluated neutrophil infiltration in skin biopsies with myeloperoxidase (MPO) and macrophage/myeloid abundance with CD68 and CD163. Among HIV-infected participants, MPO^+^ neutrophils were more abundant in lesional sites from MRSA-infected participants compared to non-lesional sites (0.16% vs. 0.006%, *P* = 0.03) and to MRSA-negative participants (0.16% vs. 0.004%, *P* = 0.001) (Fig 3D). Among HIV-uninfected participants, MPO^+^ neutrophils were also more abundant in lesional sites from MRSA-infected participants compared to non-lesional sites (0.05% vs. 0.005%, *P* = 0.006) and to MRSA-negative participants (0.01% vs. 0.05%, *P* = 0.04). Similarly, macrophage/myeloid cells (CD68^+^ or CD163^+^) were more abundant in lesional sites from HIV-infected participants compared to non-lesional sites (4.60% vs. 1.04%, *P* = 0.01) and MRSA-uninfected (4.60% vs. 0.84, *P* = 0.002) participants (Fig 3E). Among HIV-uninfected participants, macrophage/myeloid cells were also more abundant in lesional sites compared to non-lesional sites (4.43% vs. 0.90%, *P*<0.0001) and MRSA-uninfected (4.43% vs. 0.82, *P* = 0.001) participants. No significant differences were observed between HIV-infected and HIV-uninfected participants in neutrophils or macrophage/myeloid cells. Abundance of CD3, CD4, IL-17, and macrophage/myeloid cells correlated significantly with each other, whereas MPO abundance only correlated with CD3 (r = 0.46, *P* = 0.0004) and macrophage/myeloid cell (r = 0.45, *P* = 0.0005) abundance (S1 Table). In sum, T cell, neutrophil, and macrophage/myeloid migration into SSTI sites was not impaired in HIV-infected participants.

## Discussion

The factors determining increased susceptibility of HIV-infected people to MRSA SSTIs have been unclear with no specific immune defect identified to date. Studies of other human immunodeficiency states and of animal models suggest that deficiencies in IFNγ or IL-17 may predispose to *S*. *aureus* infections. Here, we show that HIV-infected participants who developed MRSA SSTIs had a lower frequency of IFNγ^+^ producing CD4 memory T cells compared to HIV-uninfected participants with MRSA SSTIs, and these IFNγ^+^ cells were less polyfunctional. We found that PBMC production of IFNγ and IL-12, which contributes to IFNγ feed-forward signaling, was lower in participants with MRSA SSTIs compared to subjects with MRSA colonization. Together, these data suggest that defective production of IFNγ, not IL-17, is the key determinant of increased susceptibility to MRSA SSTIs in HIV-infected people.

The effects of IFNγ include T-cell proliferation and survival, MHC expression on dendritic cells and macrophages, increased antigen presentation, stimulation of reactive nitrogen species by macrophages, NK cell activation and stimulation of neutrophil superoxide production and respiratory burst [11]. The mechanism by which defective IFNγ responses contributed to SSTI susceptibility is unclear but may be associated with defective neutrophil function despite effective opsonization, as people with CGD and other neutrophil disorders are also susceptible to *S*. *aureus* infections.

Whether decreased antigen-specific IFNγ production may also contribute to increased susceptibility to and recurrence of other infections is unclear. Reactivation of varicella zoster virus (VZV) and herpes simplex virus 2 (HSV-2) occurs more often in HIV-infected people [12, 13]. Defective CD8 T-cell function (resulting from inadequate CD4 T-cell signals) and NK cell and macrophage functions may account for this reactivation in the setting of HIV infection [13]. In older people, decreased frequencies of circulating VZV-specific IFNγ-producing CD4 T cells have been observed [14], and people with advanced HIV infection produced less VZV-specific IFNγ based on whole blood release assays regardless of ART [15]. Similarly, IFNγ-producing CD4 T cells protect against HSV-2 infection in immunized mice [16]. Whether decreased frequencies of IFNγ-producing CD4 T cells predispose HIV-infected people to more frequent or severe reactivation of these viruses remains to be determined.

While CD57^+^ CD4 T cells from HIV-infected persons can produce IFNγ, they have decreased proliferative capacity upon stimulation [17]. Indeed, decreased CD127 expression [18] and IL-2 production [17] may account for the decreased proliferation of CD57^+^ CD4 T cells. Higher CD57^+^ CD4 T-cell frequencies have been associated with increased development of Kaposi's sarcoma [19], which may reflect defective antigen-specific responses. Consistent with this aging CD4 T-cell phenotype, the cases were older in the Kaposi's sarcoma cohort [19], just as in this MRSA cohort. The extent to which age impacted our findings is unclear, but it is worth noting that responses to *Candida albicans*, CMV, and *Mycobacterium avium* were not impaired despite the older age of the HIV-infected participants. Therefore, higher CD57^+^ CD4 T-cell frequencies in HIV-infected participants with MRSA SSTIs relative to the HIV-uninfected participants with MRSA SSTIs may contribute to the decreased IFNγ production upon MRSA stimulation.

IFNγ production in response to *S*. *aureus* stimulation or SEB requires IL-12, which is produced by macrophages, monocytes, neutrophils and dendritic cells [20]. IL-12 upregulates IL-12Rβ2 expression and subsequently IFNγ production. HIV-infected people produce less IL-12, resulting in lower IL-12Rβ2 expression [21]. IFNγ also stimulates production of the functional IL-12 heterodimer, IL-12p70 [22]. Thus, decreased IFNγ production may account for decreased IL-12p70 release from PBMCs from HIV-infected participants with MRSA SSTIs. IL-12 deficiency is unlikely to predispose HIV-infected people to MRSA infections, as *S*. *aureus* infections are not reported in people with IL-12p40 deficiency [23] or IL-12Rβ1 deficiency [24]. However, PBMCs from people with hyperimmunoglobulin E syndrome, who are predisposed to *S*. *aureus* infections, produce less IFNγ upon stimulation with heat-killed *S*. *aureus* or with IL-12 and IL-18 but have frequencies of *Candida albicans*-specific IFNγ-producing CD4 T-cells comparable to healthy volunteers [9, 25].

IL-15 also stimulates IFNγ production and release, which further increases IL-15 production [26]. IL-15 activates NK cells, stimulates T-cell proliferation and chemotaxis and contributes to effector T-cell production. Thus, lower MRSA-induced IL-15 production by PBMCs from HIV-infected participants with MRSA SSTIs compared to HIV-uninfected participants is consistent with their lower IFNγ^+^ CD4 T-cell frequencies. These observations, together with the antigen-specific nature of IFNγ deficiency, argue for the main predisposition to be a defect in adaptive immunity.

Although CD3 and CD4 T cells were not recruited to the site of the MRSA SSTIs in all HIV-infected participants, lesion resolution was not compromised. Intact neutrophil and macrophage/myeloid cell recruitment may account for the unimpaired resolution. Dermal Th17 recruitment was not impaired in HIV-infected participants, consistent with similar homing marker expression across populations and with neutrophil recruitment. Thus, unlike in the intestinal tract [10], preferential Th17 depletion does not occur in the skin in HIV-infected people. Rather, intact neutrophil recruitment is consistent with the finding that HIV-infected people resolve acute MRSA SSTIs as effectively as HIV-uninfected people [27].

IFNγ administration has been used to prevent *S*. *aureus* infections. IFNγ decreases the frequency and severity of *S*. *aureus* infections in people with CGD [7]. In contrast, despite the increased predisposition to *S*. *aureus* infections, HIV-infected persons do not appear to be at increased risk for severe manifestations such as septic shock [28]. In a study performed over 25 years ago, no people with AIDS who received IFNγ therapy developed *S*. *aureus* bacteremia or nonbacteremic infections, compared to 5 of 52 who received IL-2 [29]. IL-2 likely did not increase the risk for these infections as *S*. *aureus* infections were not reported in ESPRIT or SILCAAT [30]. Whether administration of IFNγ, or its drivers IL-12 and IL-15, would prevent *S*. *aureus* infections in larger cohorts of HIV-infected people remains unknown. In addition, the effects of early ART with HIV suppression on MRSA-specific responses warrant study.

Taken together, our data suggest that deficient IFNγ production by MRSA-specific CD4 T cells predisposes HIV-infected people to MRSA SSTIs. The decreased IFNγ production is not associated with defective phagocytosis or decreased neutrophil homing to the skin. Thus, eliciting MRSA-specific IFNγ responses may be essential for effective preventive strategies, including vaccines, and novel therapeutics against MRSA SSTIs.

## Materials and Methods

### Study design

We conducted a prospective study among HIV-infected adults at the Naval Medical Center San Diego and Walter Reed Army Medical Center. All participants in the study were at least 18 years of age and were military beneficiaries. HIV-infected persons had positive ELISA and confirmatory Western Blot testing, while the HIV-uninfected group had negative HIV ELISA testing. “MRSA SSTIs” were defined as culture-confirmed cellulitis, furuncles, carbuncles or abscesses on any part of the body that were not related to an indwelling intravenous catheter or a surgical procedure, and these participants were enrolled within 7 days of presentation. "MRSA colonized" was defined as the presence of MRSA in the nares, axilla or groin without evidence of ongoing infection or infection in the past year, and these participants were enrolled within 14 days of their most recent positive colonization result. "MRSA-negative" was defined as participants with one swab each of nares, axilla and groin (a swab collected at each of these sites) that tested negative for MRSA within 14 days of enrollment. PBMCs were cryopreserved at enrollment. PBMCs from before enrollment (T1, median 244 days, range 133–391 days) and after enrollment (T3, median 237 days, range 140–404 days) were available from the HIV-infected participants from the Natural History Study (NHS) Repository (RV168 NHS study, IDCRP-000). No longitudinal samples were available from HIV-uninfected participants.

### Intracellular cytokine assay

Blood was collected from each participant at the time of enrollment, processed by Ficoll density-gradient centrifugation, minimizing the time blood contacts the Ficoll. Peripheral blood mononuclear cells (PBMCs) were resuspended in prechilled 10% DMSO + 90% heat inactivated, filtered fetal calf serum (Gibco, ThermoFisher Scientific, Inc, Waltham, MA) and transferred to a pre-chilled isopropanol bath (Mr. Frosty by Nalgene, ThermoFisher Scientific, Inc, Waltham, MA). PBMCs were stored at -80°C overnight before transferring to liquid nitrogen. PBMCs were thawed using pre-warmed RPMI medium with 10% heat-inactivated fetal calf serum (Gibco), washed twice, and rested for two hours prior to stimulation. Stimulation was performed on PBMCs resuspended at 5x10^6^/ml in 200 μl RPMI medium with 10% heat-inactivated fetal calf serum (Gibco) and with 1 μg/ml anti-CD28 and anti-CD49d (BD) antibodies.

For generating heat-killed MRSA, 5 ml of Trypsin Soy Broth (TSB) was inoculated with one colony of USA300 *S*. *aureus* (courtesy of Frank DeLeo, Rocky Mountain Labs) and incubated overnight at 37°C. 100 μL of culture was diluted into 10mL of TSB. After 3 hours, the OD600 was measured and contrasted against a known absorbable growth curve. Bacteria were washed twice and resuspended to 10^9^ colony-forming units per ml of PBS. Bacteria were incubated at 65°C for 45 minutes. Sterility was confirmed by no growth on blood agar plates at 48 hours.

Two sets of cultures were set up for each condition: one set with 10^6^ PBMCs and one set with 10^5^ PBMCs. All PBMCs were stimulated with heat-killed MRSA, *Candida albicans* cellular antigen (Greer), cytomegalovirus pp65, *Staphylococcus* enterotoxin B (SEB, Sigma), or *Mycobacterium avium* purified protein derivative (Statens Serum Institute) with anti-CD28 and anti-CD49d antibodies, or anti-CD28 and anti-CD49d antibodies alone. After 2 hours of stimulation at 37°C, brefeldin A (1 μg/ml) was added to the set with 10^6^ PBMCs and both sets of PBMCs were stimulated for an additional 14 hours at 37°C. Cell culture supernatants were aspirated from the set with 10^5^ PBMCs after centrifugation and frozen. The other set of PBMCs was incubated with viability dye (LIVE/DEAD Fixable Aqua Dead Cell Stain Kit, Life Technologies, Grand Island, NY), surface stained with phenotypic markers and stained intracellularly with cytokines of interest. The frequency of cytokine-positive cells is presented as a percentage of memory (CD27^+^CD45RO^+^ or CD27^-^) cells. All experiments were performed blinded to the HIV and MRSA status of each study participant.

### Flow cytometry and multiplex cytokine assays of cell culture supernatant

Eighteen-parameter flow cytometry analysis was performed with an LSR II (BD Biosciences, San Jose, CA). CD3 H7 allophycocyanin (APC, BD Biosciences) or CD40 ligand (APC-eFluor 780, eBioscience), CD3 Qdot655, CD4 Cy5.5 phycoerythrin (PE, Invitrogen), CD8 Qd705 (Invitrogen), CD27 PC5 (Beckman Coulter), CD45RO ECD (Beckman Coulter), CD14 Pacific Blue (BD Biosciences), CD57 Qdot655 (BD Biosciences), IL-17 PE (eBioscience) or IL-10 PE (BD Biosciences), IL-17 fluorescein isothiocyanate (FITC, eBioscience) or IL-1β FITC (eBioscience), IL-22 APC (R&D Systems) or IL-2 APC (BD Biosciences), IFNγ Cy7PE (BD Biosciences) and TNF Alexa 680 (BD Biosciences) were used in two separate panels. The data were analyzed using FlowJo (Tree Star), PESTLE (Mario Roederer) and SPICE (Mario Roederer). The values reported are background-subtracted.

Multiplex cytokine assays were performed using a customized Bio-Plex Pro assay (Bio-Rad) according to the manufacturer’s instructions and analyzed on a Luminex 200 System. The following cytokines were measured: IL-1β, IL-2, IL-4, IL-5, IL-6, IL-7, IL-8, IL-10, IL-12 (p70), IL-13, IL-15, IL-17, G-CSF, GM-CSF, IFNγ, MCP-1, MIP-1β and TNF.

### Amnis Imagestream

Freshly thawed PBMCs were resuspended at 5x10^6^/ml in 200 μl RPMI medium with 10% heat-inactivated fetal calf serum (Gibco). PBMCs were pelleted and resuspended in 1:100 *S*. *aureus* pHrodo (Life Technologies) or DMEM and incubated at 37°C for 3 hours. PBMCs were washed and labeled with CD3 Pacific Blue, CD20 V450, CD56 V450 (BD Biosciences), and violet viability (LIVE/DEAD Fixable Violet Viability/Vitality Kit, Life Technologies), for a discard channel; CD14 FITC, and CD16 Cy5PE (BD Biosciences). Data were acquired using the ImageStream (Amnis Corporation, Seattle, WA) and analyzed with IDEAS (Amnis Corporation). Cells were gated on forward and side scatter, and CD3^+^, CD20^+^, CD56^+^, CD16^+^ and dead cells were excluded.

### Opsonization assay

*Staphylococcus aureus* BioParticles conjugated to AlexaFluor 488 (Molecular Probes, Eugene, OR) were resuspended in PBS at 20 mg/ml and incubated in 20% participant plasma at 37°C for 1 hour. The bacteria were diluted 1:10 (v/v) in HBSS with calcium/magnesium. Neutrophils from a healthy donor (Astarte Biologics, Bothell, WA) were thawed in HBSS with calcium/magnesium and warmed at 37°C for 10 minutes. 5x10^5^ neutrophils were incubated with 5x10^5^ opsonized bacteria at 37°C for 10 minutes while rotating gently. Cells were placed on ice, fixed with cold 4% paraformaldehyde, and resuspended in HBSS (no calcium/magnesium). Fluorescence was measured on a Fortessa (BD Biosciences, San Jose, CA).

### Histological evaluation

Skin biopsies were optional and performed within 3 days of blood sampling. Skin biopsies consisted of a single 0.60 cm punch biopsy from the edge of the MRSA SSTI and a single 0.60 cm punch biopsy from a distant uninfected site for those with MRSA infections. Participants colonized or negative for MRSA underwent skin biopsy at a random body site. Tissue was fixed in zinc formalin.

Immunohistochemistry was performed using a biotin-free polymer approach (Golden Bridge International, Inc.) on 5-μm tissue sections mounted on glass slides, which were dewaxed and rehydrated with double-distilled H_2_O. The multi-staining CD4/CD68/CD163 to quantify CD4 T cells was performed as describe previously [31]. The intense staining of CD68 and CD163 masks the faint CD4 on myeloid lineage cells to facilitate CD4 T-cell identification. Heat induced epitope retrieval (HIER) was performed by heating sections in 10 mM Citrate (pH 6.0) or 1x Diva retrieval buffer (Biocare Medical) in a pressure cooker (Decloaking Chamber model DC2002; Biocare Medical) set at 122–125°C for 30 sec. Slides were incubated with blocking buffer (TBS with 0.05% Tween-20 and 0.5% casein) for 10 min and then incubated with mouse anti-CD68 (1:400; clone KP1, Dako), mouse anti-CD163 (1:400; clone10D6; Novocastra/Leica) and rabbit monoclonal anti-CD4 (1:200; clone EPR6855; Epitomics, Inc.) or mouse monoclonal anti-CD3 (1:100; Dako; clone F7.2.38), rabbit polyclonal anti-myeloperoxidase (1:1,000; Dako; Cat. No. A0398), or goat anti-IL17 (1:100; R&D Systems; Cat. No. AF-317-NA) diluted in blocking buffer and at room temperature for 1 h or overnight at 4°C. Slides were washed in 1x TBS with 0.05% Tween-20, endogenous peroxidases were blocked using 1.5% (v/v) H_2_O_2_ in TBS (pH 7.4) for 10 min, and slides were incubated with Mouse or Goat Polink-2 HRP (Golden Bridge International, Inc.) or Rabbit Polink-1 HRP according to manufacturer’s recommendations. Slides were developed with Warp Red (Biocare Medical, Inc.) to develop macrophage/myeloid cells or Impact DAB (3,3′-diaminobenzidine; Vector Laboratories) for the remaining antibodies. Slides were washed in ddH_2_O, counterstained with hematoxylin, mounted in Permount (Fisher Scientific), and scanned at high magnification (x200) using the ScanScope CS System (Aperio Technologies), yielding high-resolution data from the entire tissue section. Representative regions of interest (ROIs; 0.25 mm^2^) were identified, and high-resolution images extracted from these whole-tissue scans. The percent area of the dermis tissue that stained for CD3 or CD4 T cells, MPO^+^ neutrophils, IL17^+^, or CD68^+^ or CD163^+^ cells was quantified using Photoshop CS5 and Fovea software.

### Ethics statement

The study was approved by the central institutional review board (IRB) at USUHS and by IRBs at the Naval Medical Center San Diego and Walter Reed Army Medical Center. All participants were at least 18 years of age and signed written informed consent before enrollment.

### Statistics

Between group comparisons were performed using the Mann-Whitney *U* test, and within group longitudinal comparisons were performed using the Wilcoxon test. As this was a hypothesis-generating study and as the pathways determining cytokine production overlap, no correction for multiple comparisons was made. Statistically significant comparisons are reported. Nonparametric Spearman correlations were calculated. All analyses were performed using Prism 6.0c software (GraphPad Software, Inc.).

## Supporting Information

S1 Fig Memory CD4 T cells in HIV-infected participants based on flow cytometry. (A) Central memory-like (CD27^+^CD45RO^+^), (B) effector memory-like (CD27^-^) and (C) terminally differentiated CD57^+^ memory CD4 T cells are shown. *P*-values were calculated using the Mann-Whitney *U* test.
(PDF)

S2 Fig Polyfunctionality of MRSA-specific memory CD4 T cells. HIV-infected participants with MRSA SSTI were compared to no MRSA SSTI or colonization based on flow cytometry data. (A) Pie chart of distribution of the frequencies of expressing 5, 4, 3, 2 or 1 of the following markers: CD40L, IL-2, TNF, IL-17, and IFNγ. (B) The frequency of MRSA-specific memory CD4 T cells expressing IFNγ, TNF and/or IL-2. *P*-values were calculated using the Wilcoxon signed rank test using SPICE.
(PDF)

S3 Fig Antigen-specific IFNγ^+^ memory CD4 T cells. Frequency of antigen-specific IFNγ^+^ memory (CD27^+^CD45RO^+^ or CD27^-^) CD4 T-cell responses in HIV-infected participants with MRSA SSTI, colonization or neither, or HIV-uninfected participants with MRSA SSTI, colonization or neither. Number per group varies based on final cell count after thawing. Thawed PBMCs were stimulated overnight with the following in the presence of brefeldin A and evaluated using multi-parameter flow cytometry: (A) CMV pp65, (B) *Candida albicans* cellular antigen (C) *Mycobacterium avium* purified protein derivative, (D) *S*. *aureus* enterotoxin B. *P*-values were calculated using the Mann-Whitney *U* test.
(PDF)

S4 Fig Change in MRSA-specific IFNγ^+^ memory or CCR4^+^ memory CD4 T cells. Frequency of MRSA-specific (A-C) IFNγ^+^ memory (CD27^+^CD45RO^+^ or CD27^-^) CD4 T cells or (D-F) CCR4^+^ memory CD4 T cells at T1 (median 244 days prior to SSTI and/or time of biopsy), T2 (time of biopsy), and T3 (median 237 days after SSTI and/or time of biopsy) in (A, D) HIV-infected MRSA SSTI, (B, E) HIV-infected MRSA colonized, and (C,F) HIV-infected MRSA negative groups. PBMCs were stimulated overnight with heat-killed MRSA in the presence of brefeldin A and evaluated by multi-parameter flow cytometry. *P*-values were calculated using the Wilcoxon matched-pairs signed rank test.
(PDF)

S5 Fig Monocytes in MRSA infection. PBMCs were stimulated overnight with heat-killed MRSA in the presence of brefeldin A and evaluated by multi-parameter flow cytometry. (A) Frequency of CD3^-^CD14^+^ cells. (B) Frequency of IL-1β^+^CD14^+^ cells. (C) Frequency of TNF^+^CD14^+^ cells. *P*-values were calculated using the Mann-Whitney *U* test.
(PDF)

S6 Fig Phagocytosis. (A-C) PBMCs were thawed and incubated with *S*. *aureus* pHrodo for 3 hours prior to surface antibody staining and evaluation by the Amnis ImageStream. (A) One *S*. *aureus* bacterium on the outside of a CD14^+^ cell (left), one *S*. *aureus* bacterium inside a CD14^+^ cell (middle), three *S*. *aureus* bacteria inside one CD14^+^ cell (right). (B) The percentage of CD14^+^ cells with internalized *S*. *aureus* bacteria in participants with HIV infection and MRSA SSTI, without HIV infection but with MRSA SSTI or neither HIV nor MRSA infection. (C) The percentage of CD14^+^ cells with 2 or more internalized *S*. *aureus* bacteria in participants with HIV infection and MRSA SSTI, without HIV infection but with MRSA SSTI or neither HIV nor MRSA infection. (D) The median fluorescent intensity of Ax488-labeled *S*. *aureus* after opsonization by participant plasma and incubation with healthy neutrophils. *P*-values were calculated using the Mann-Whitney *U* test.
(PDF)

S1 Table Correlations among abundance of CD3, CD4, IL-17, myeloid (CD68 and/or CD163), and myeloperoxidase (MPO) in skin biopsies. (DOCX)
