# Supplementary figures and images for: MRSA Infections in HIV-Infected People Are Associated with Decreased MRSA-Specific Th1 Immunity
Source: PLoS Pathog. 2016 Apr 19;12(4):e1005580. doi: 10.1371/journal.ppat.1005580 (PMC4836670; doi:10.1371/journal.ppat.1005580)

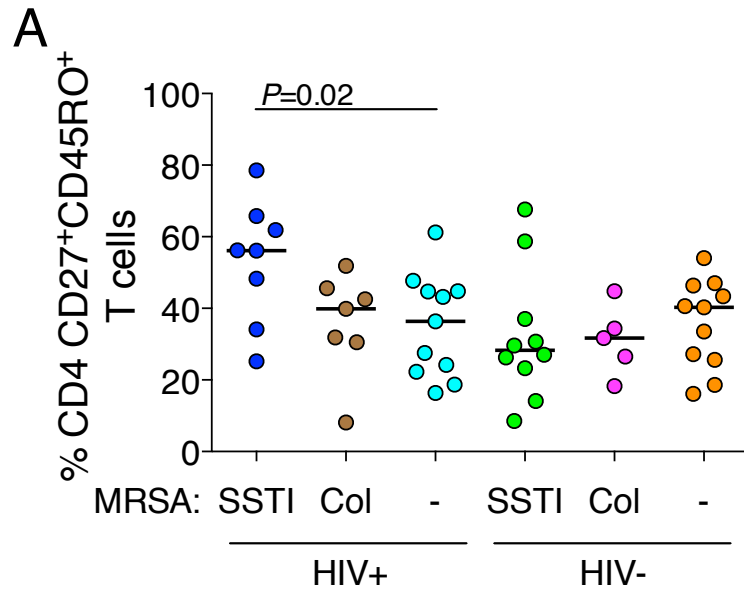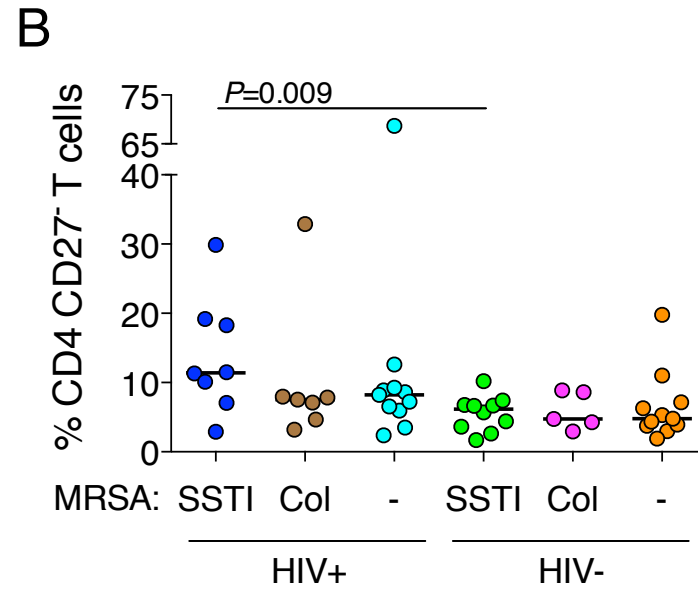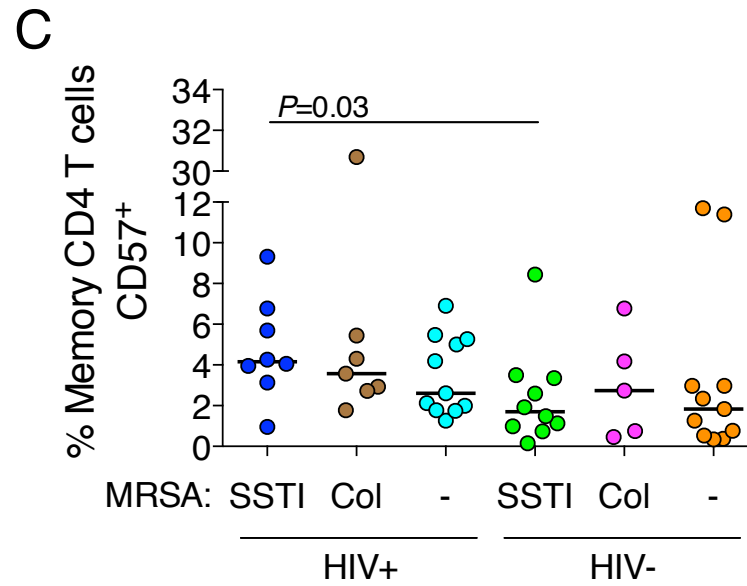

Supplement: S1 Fig — (A) Central memory-like (CD27+CD45RO+), (B) effector memory-like (CD27-) and (C) terminally differentiated CD57+ memory CD4 T cells are shown. P-values were calculated using the Mann-Whitney U test. (PDF) [file ppat.1005580.s001.pdf]

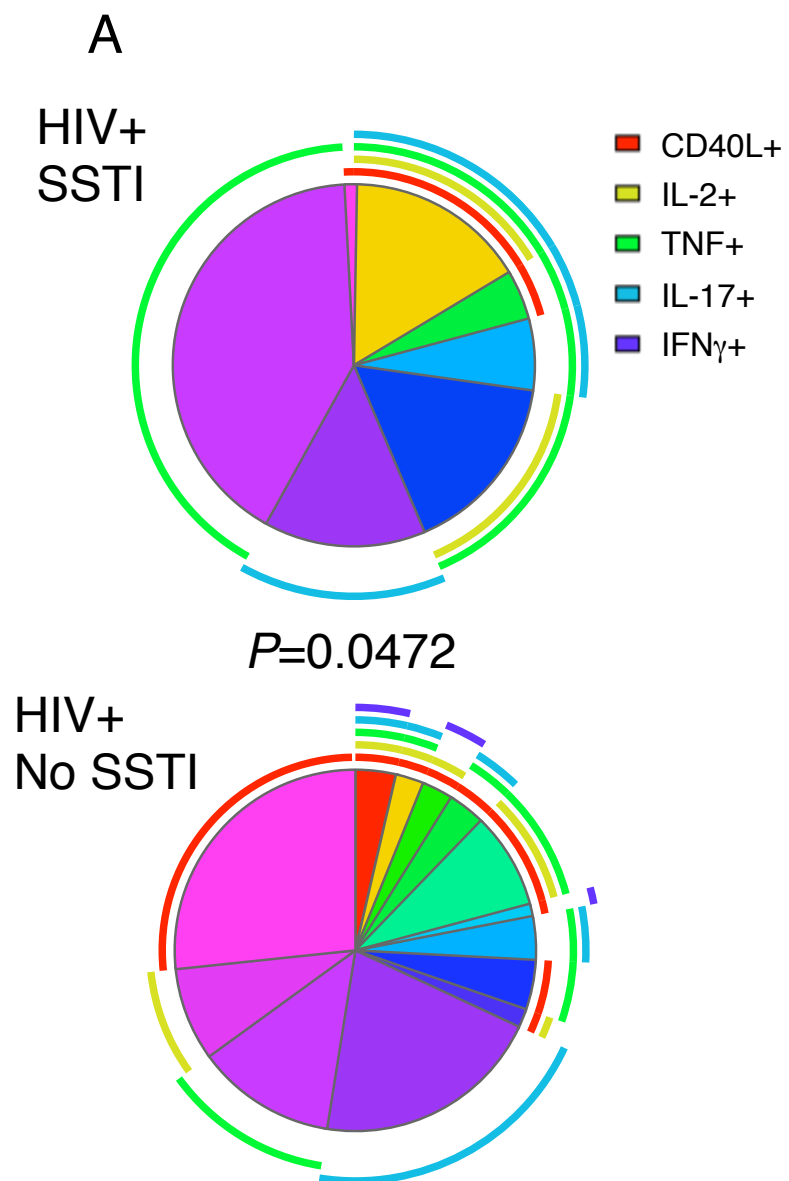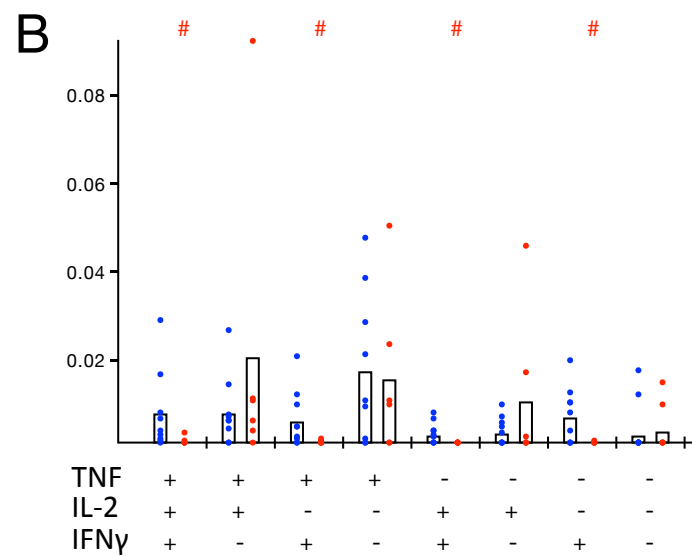

| TNF | IL-2 | IFN $\gamma$ | <i>P</i> |
|-----|------|--------------|----------|
| +   | +    | +            | 0.02     |
| +   | +    | -            | 0.77     |
| +   | -    | +            | 0.03     |
| +   | -    | -            | 0.91     |
| -   | +    | +            | 0.01     |
| -   | +    | -            | 0.95     |
| -   | -    | +            | 0.05     |
| -   | -    | -            | 0.81     |

Supplement: S2 Fig — HIV-infected participants with MRSA SSTI were compared to no MRSA SSTI or colonization based on flow cytometry data. (A) Pie chart of distribution of the frequencies of expressing 5, 4, 3, 2 or 1 of the following markers: CD40L, IL-2, TNF, IL-17, and IFNγ. (B) The frequency of MRSA-specific memory CD4 T cells expressing IFNγ, TNF and/or IL-2. P-values were calculated using the Wilcoxon signed rank test using SPICE. (PDF) [file ppat.1005580.s002.pdf]

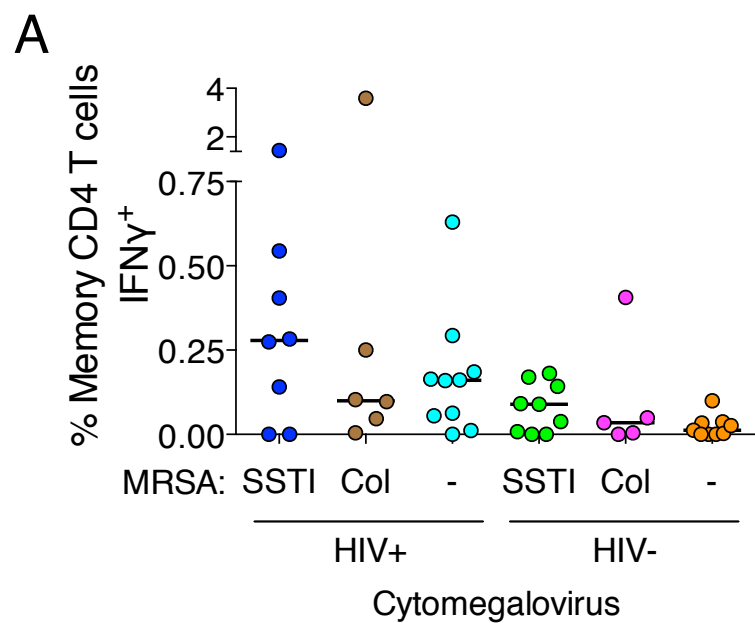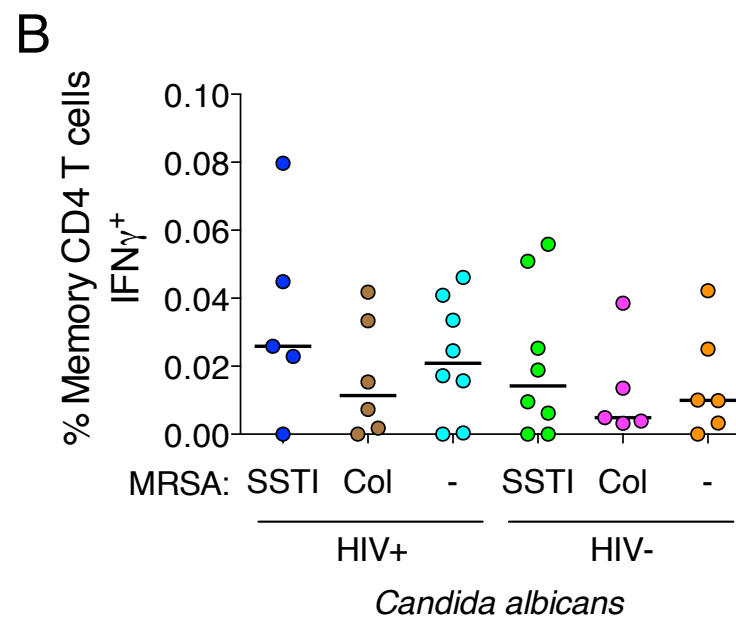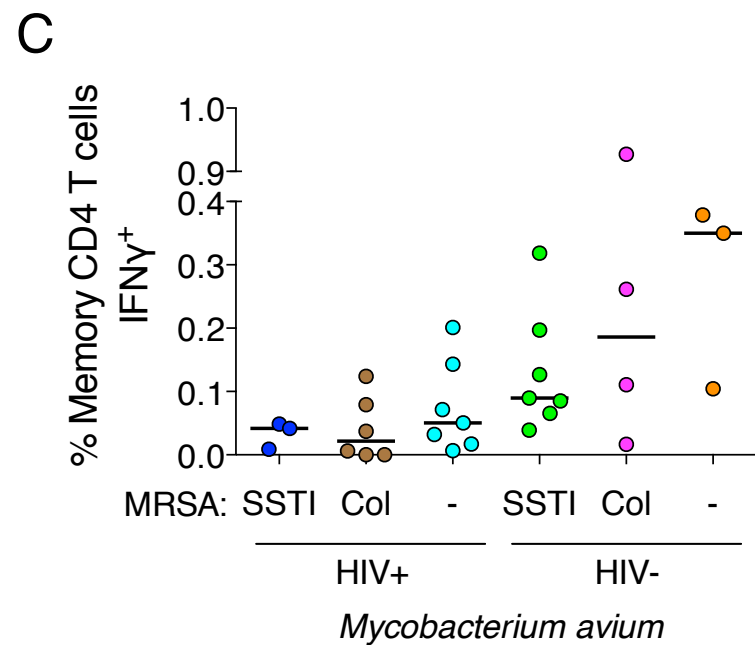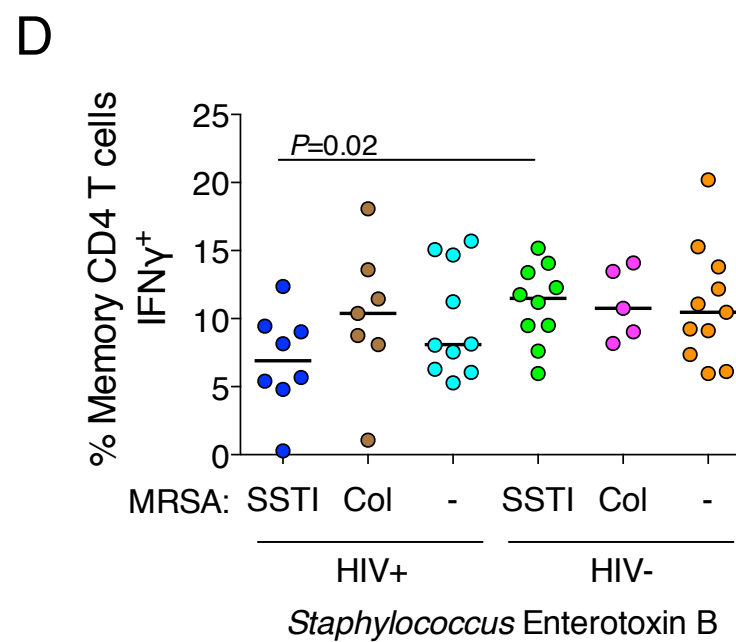

Supplement: S3 Fig — Frequency of antigen-specific IFNγ+ memory (CD27+CD45RO+ or CD27-) CD4 T-cell responses in HIV-infected participants with MRSA SSTI, colonization or neither, or HIV-uninfected participants with MRSA SSTI, colonization or neither. Number per group varies based on final cell count after thawing. Thawed PBMCs were stimulated overnight with the following in the presence of brefeldin A and evaluated using multi-parameter flow cytometry: (A) CMV pp65, (B) Candida albicans cellular antigen (C) Mycobacterium avium purified protein derivative, (D) S. aureus enterotoxin B. P-values were calculated using the Mann-Whitney U test. (PDF) [file ppat.1005580.s003.pdf]

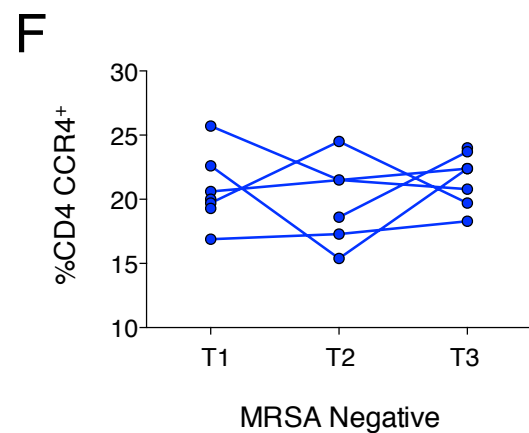

Supplement: S4 Fig — Frequency of MRSA-specific (A-C) IFNγ+ memory (CD27+CD45RO+ or CD27-) CD4 T cells or (D-F) CCR4+ memory CD4 T cells at T1 (median 244 days prior to SSTI and/or time of biopsy), T2 (time of biopsy), and T3 (median 237 days after SSTI and/or time of biopsy) in (A, D) HIV-infected MRSA SSTI, (B, E) HIV-infected MRSA colonized, and (C,F) HIV-infected MRSA negative groups. PBMCs were stimulated overnight with heat-killed MRSA in the presence of brefeldin A and evaluated by multi-parameter flow cytometry. P-values were calculated using the Wilcoxon matched-pairs signed rank test. (PDF) [file ppat.1005580.s004.pdf]

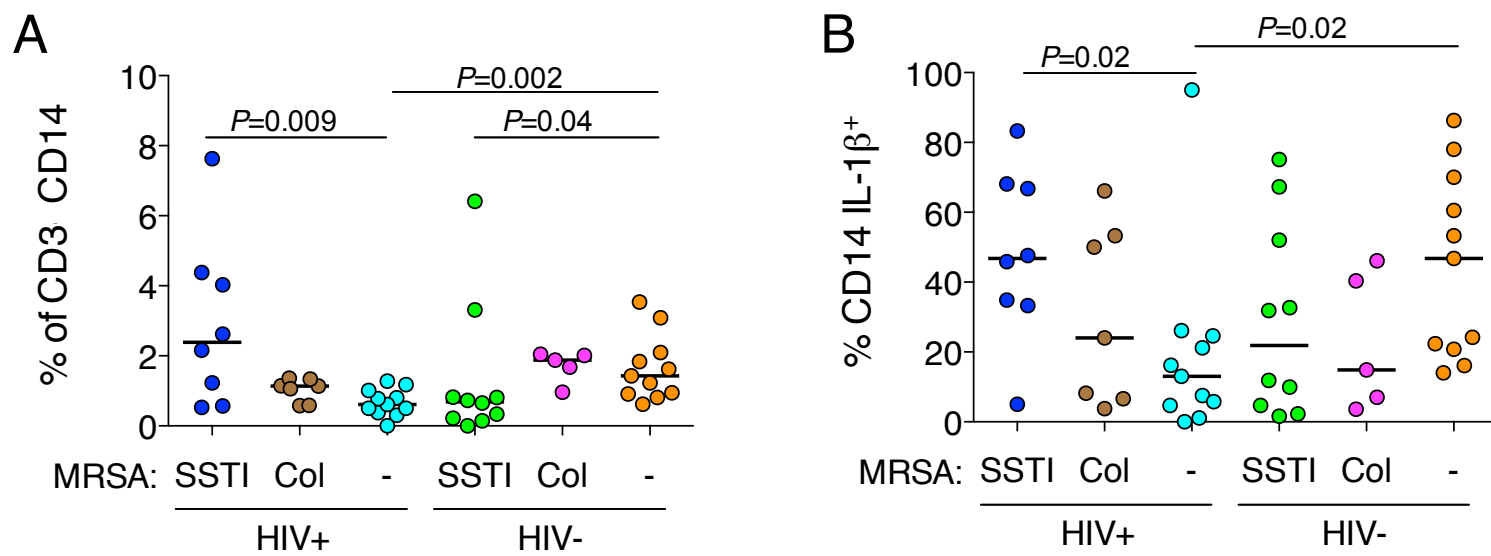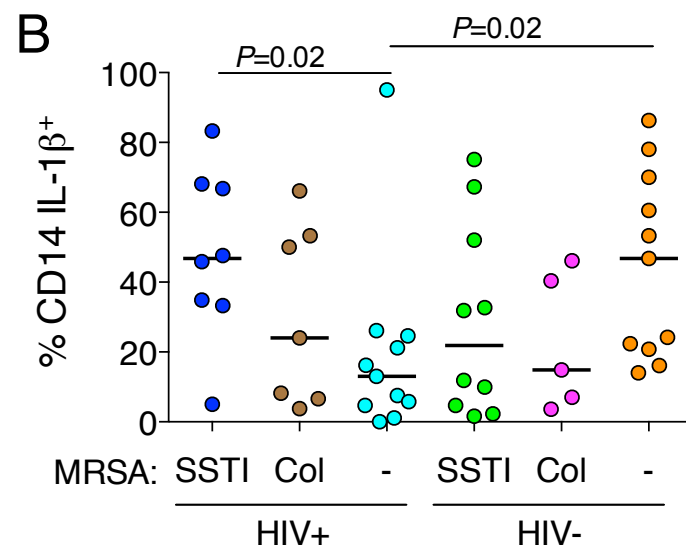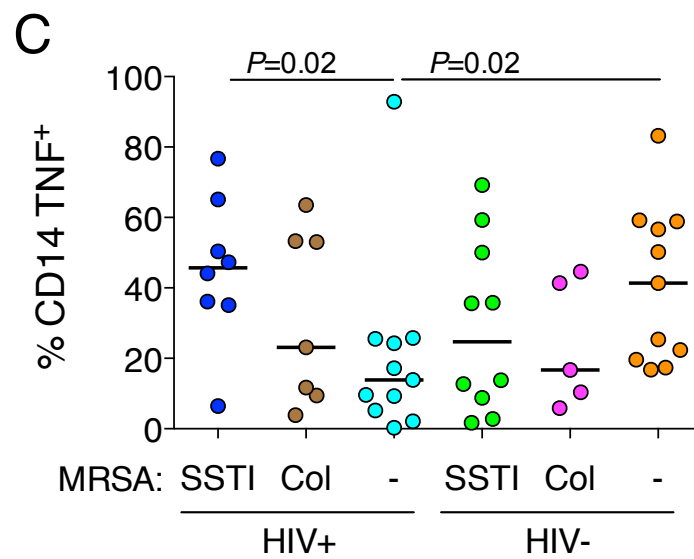

Supplement: S5 Fig — PBMCs were stimulated overnight with heat-killed MRSA in the presence of brefeldin A and evaluated by multi-parameter flow cytometry. (A) Frequency of CD3-CD14+ cells. (B) Frequency of IL-1β+CD14+ cells. (C) Frequency of TNF+CD14+ cells. P-values were calculated using the Mann-Whitney U test. (PDF) [file ppat.1005580.s005.pdf]

A

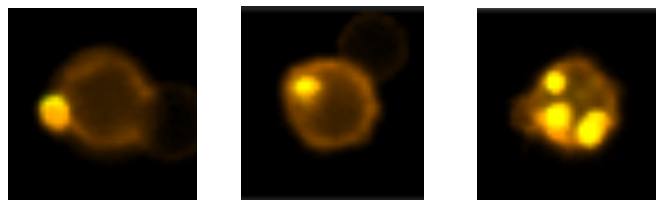

B

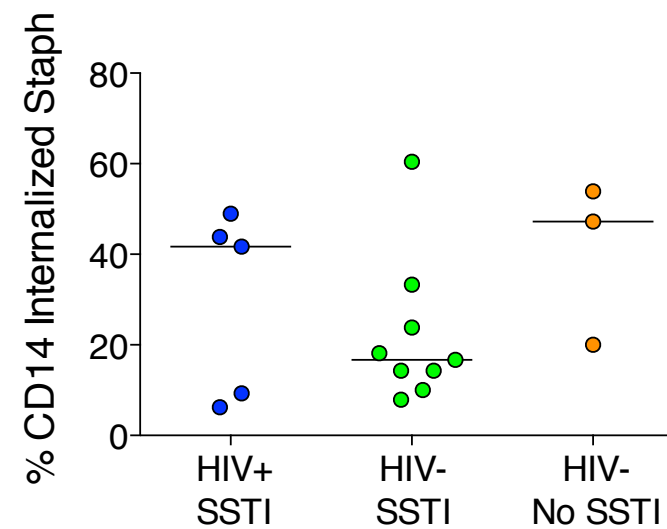

C

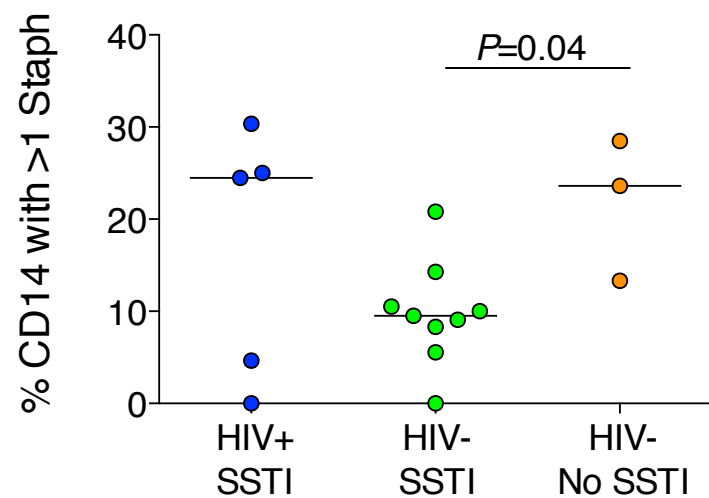

D

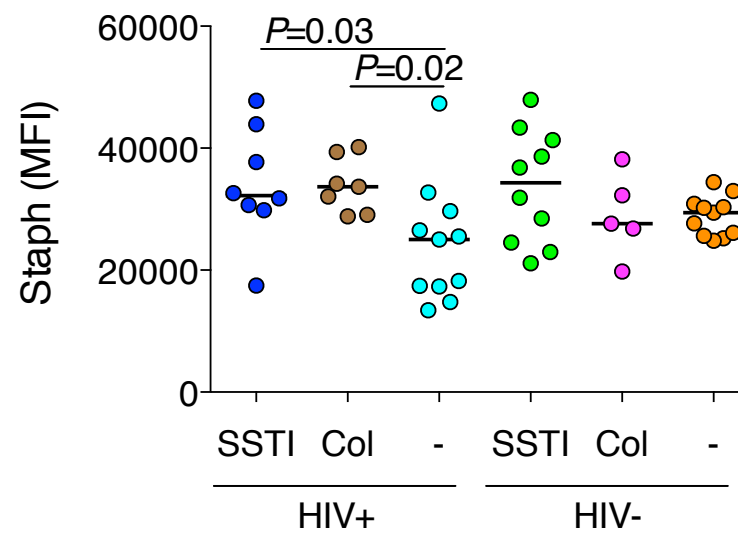

Supplement: S6 Fig — (A-C) PBMCs were thawed and incubated with S. aureus pHrodo for 3 hours prior to surface antibody staining and evaluation by the Amnis ImageStream. (A) One S. aureus bacterium on the outside of a CD14+ cell (left), one S. aureus bacterium inside a CD14+ cell (middle), three S. aureus bacteria inside one CD14+ cell (right). (B) The percentage of CD14+ cells with internalized S. aureus bacteria in participants with HIV infection and MRSA SSTI, without HIV infection but with MRSA SSTI or neither HIV nor MRSA infection. (C) The percentage of CD14+ cells with 2 or more internalized S. aureus bacteria in participants with HIV infection and MRSA SSTI, without HIV infection but with MRSA SSTI or neither HIV nor MRSA infection. (D) The median fluorescent intensity of Ax488-labeled S. aureus after opsonization by participant plasma and incubation with healthy neutrophils. P-values were calculated using the Mann-Whitney U test. (PDF) [file ppat.1005580.s006.pdf]
