# Supplementary material for: MRSA Infections in HIV-Infected People Are Associated with Decreased MRSA-Specific Th1 Immunity
Source: PLoS Pathog. 2016 Apr 19;12(4):e1005580. doi: 10.1371/journal.ppat.1005580 (PMC4836670; doi:10.1371/journal.ppat.1005580)
Supplement: S1 Table — (DOCX) [file ppat.1005580.s007.docx]

|  | CD3 | CD4 | CD68 | IL-17 | MPO |
| --- | --- | --- | --- | --- | --- |
| CD3 |  | r=0.54  *P*<0.0001 | r=0.62  *P*<0.0001 | r=0.43  *P*=0.01 | r=0.46  *P*=0.0004 |
| CD4 | r=0.54  *P*<0.0001 |  | r=0.58  *P*<0.0001 | r=0.38  *P*=0.03 | r=0.21  *P*=n.s. |
| CD68 | r=0.62  *P*<0.0001 | r=0.58  *P*<0.0001 |  | r=0.68  *P*<0.0001 | r=0.45  *P*=0.0005 |
| IL-17 | r=0.43  *P*=0.01 | r=0.38  *P*=0.03 | r=0.68  *P*<0.0001 |  | r=0.33  *P*=n.s. |
| MPO | r=0.46  *P*=0.0004 | r=0.21  *P*=n.s. | r=0.45  *P*=0.0005 | r=0.33  *P*=n.s. |  |
